# Statistical Reasoning: Choosing and Checking the Ingredients, Inferences Based on a Measure of Statistical Evidence with Some Applications

**DOI:** 10.3390/e20040289

**Published:** 2018-04-16

**Authors:** Luai Al-Labadi, Zeynep Baskurt, Michael Evans

**Affiliations:** 1 Department of Mathematics, University of Sharjah, P.O. Box 27272 Sharjah, United Arab Emirates; 2 Genetics and Genome Biology, Hospital for Sick Children, Toronto, ON M5G 1X8, Canada; 3 Department of Statistical Sciences, University of Toronto, Toronto, ON M5S 3G3, Canada

**Keywords:** statistical reasoning, model checking, elicitation of priors, checking priors, measuring statistical evidence, relative belief inferences

## Abstract

The features of a logically sound approach to a theory of statistical reasoning are discussed. A particular approach that satisfies these criteria is reviewed. This is seen to involve selection of a model, model checking, elicitation of a prior, checking the prior for bias, checking for prior-data conflict and estimation and hypothesis assessment inferences based on a measure of evidence. A long-standing anomalous example is resolved by this approach to inference and an application is made to a practical problem of considerable importance, which, among other novel aspects of the analysis, involves the development of a relevant elicitation algorithm.

## 1. Introduction

It is relevant to ask what characteristics should be required of a theory of statistical reasoning. The phrase *statistical reasoning* is used here, as opposed to statistical inference, because there is a logical separation between how the ingredients to a statistical problem are chosen and checked for their validity, and the inference step that involves the application of the rules of a theory of inference to the ingredients. Thus, it is argued in Section 2 that there are two aspects to a theory of statistical reasoning: (i) specifying methodology for choosing and checking the ingredients to a statistical analysis beyond the data and (ii) specifying a theory of inference using these ingredients that is based on a measure of statistical evidence. These components correspond to the premises and the argument in logical reasoning.

In Section 3, a specific theory of statistical reasoning, as described in [1], that satisfies the criteria developed in Section 2 is reviewed. It is shown that an application of the theory of relative belief inference resolves difficulties in a problem that has led to anomalous results for other theories. It is to be noted that a number of additional examples have been published demonstrating that relative belief inference can lead to more satisfactory results than other approaches (see [2,3,4,5,6]). Particularly noteworthy is the Jeffreys–Lindley paradox where an increasingly diffuse prior typically leads to overwhelming evidence in favor of a hypothesis even when it seems contradicted by the data. The discussion of this paradox in [1] shows that the relative belief approach to inference leads to a clear resolution. A goal of this paper is to argue in favor of the relative belief approach to statistical inference based on its logical coherence and its utility in applications.

In Section 4, the theory of statistical reasoning is applied to an important practical problem where some inferential difficulties have arisen, namely, the problem of determining whether or not there is a relationship between a binary-valued response variable and predictors. For this, response y∈{0,1} is related to *k* quantitative predictors $x=(x_{1},\ldots ,x_{k})$ via y∼ Bernoulli(p(x)) with

(1) $$p\left(x\right)=G\left(\beta_{1}x_{1}+\cdots +\beta_{k}x_{k}\right),$$

where *G* is a known cdf and $\beta =(\beta_{1},\ldots ,$ $\beta_{k})\in R^{k}$ is unknown. This can be regarded as a case-study for the overall approach and a number of novel results are obtained. Perhaps the biggest challenge with this model is determining a suitable prior and in Section 4.1 an elicitation algorithm is developed that improves on earlier efforts. The bias in the prior is measured in Section 4.2. Model checking is essential, namely, determining if (1) holds at least approximately. Since this is dealt with in [7], this approach is used here without much discussion. The check for prior-data conflict is developed in Section 4.3, together with an approach to modifying the prior when necessary, and relative belief inferences are applied in Section 4.4.

The main theme of the approach discussed here for (ii) centers around being clear about how statistical evidence is to be measured and, as will be seen, this has implications for (i) as well. Certainly, the concept of statistical evidence plays a key role in the development of the subject, but, as discussed in Chapter 3 of [1], it can be argued that there is a general failure of most approaches to deal adequately with this concept. There have, however, been a number of measures of evidence proposed in the philosophical and statistical literature and many of these are related to the relative belief ratio. For example, the Bayes factor is one such measure, but the relative belief ratio can be considered as more basic (see Section 3), and this comment applies to a number of other measures as well as discussed in Chapter 4 of [1]. The relative belief ratio is certainly not a new measure of evidence as [8] refers to the log of this quantity as the information and there are some references to Keynes calling it the association factor. As far as we know, however, there have been no attempts to use this quantity as the central concept in derivation of a theory of statistical inference. This leads to a number of unique characteristics for the theory outlined in Section 3.

There are many others who have also focused on treating statistical evidence and its measurement as a central concept in the subject. Jeffreys’ earliest papers focus on this (see also [9]), and the paper [10] provides excellent coverage of this history. In addition, the paper [11] summarizes developments by these authors that is certainly in the same spirit as what is being advocated here for (ii). The philosophy of science literature on the topic of evidence is vast and we point to [12] as a nice introduction with [13] being a more detailed discussion. The work of [14] is also particularly noteworthy in this regard.

## 2. The Foundations of Statistical Reasoning

When concerned with the foundations of statistics, it is reasonable to ask: what is the purpose of statistics as a subject or what is its role in science? To answer this, consider a context where an investigator has interest in some quantity and either wants to know (E) the value of this quantity or has a theory that leads to a specific value for the quantity and so wants to know (H) if this value is indeed correct and so test the theory. In addition, the investigator has available data d, produced in some fashion, which it is believed contains *evidence* concerning answers to (E) and (H). The purpose of statistical theory is to provide a reasoning process that can be applied to *d* to determine what the evidence has to say about (E) or (H), namely, produce an estimate of the quantity based on the evidence or assess whether there is evidence either in favor of or against the hypothesized value. In addition, as is widely recognized, estimation and hypothesis assessment should also produce a measure of the accuracy of the estimate and a measure of the strength of the evidence for or against the hypothesis. Answering (E) and/or (H) is called statistical inference and a sound, logical theory of statistical inference, which contained the minimal ingredients possible, can be viewed as a major goal of the subject.

Any theory that does not lead to specific answers to (E) and (H) or is dependent on ingredients or rules of reasoning that are not well-justified is unsatisfactory. In the end, the believability of the inferences drawn is entirely dependent on the soundness of the theory that produced them. Thus, statistics is not an empirical subject like physics, where conclusions can also be assessed against the empirical world, but is more like an extension of purely logical reasoning to contexts where the data does not lead to categorical answers to (E) and (H) and so produces uncertainty. The view is taken here that we want to maintain a close relationship between a theory of statistical reasoning and the theory of logical reasoning. This has a number of consequences with perhaps the most important being that it implies a separation of the assessment of the appropriateness of the ingredients specified to a statistical analysis beyond the data, and the theory producing the inferences. The ingredients play the role of the premises and the theory of statistical inference takes the role of the rules of inference used in a logical argument. The separation of these aspects of a logical argument has been understood since Aristotle (see [15]).

There are two main theses of the argument developed here: (I) all ingredients to a statistical analysis must be checkable against the data and (II) the theory of inference must be based on a measure of statistical evidence. The rationale for (I) and (II) are now considered with (II) discussed first, as it plays a key role.

The concept of the evidence in the data is clearly of utmost importance to statistical reasoning. There is no need, however, to provide a measure of the total evidence contained in the data, for the measure of evidence only has to deal with (E) and (H) for the quantity of interest. The measure of evidence must clearly indicate whether there is evidence for or against any specific value of the quantity of interest being the true value. This follows from the desired relationship with logic, as the rules of logical inference assume the truth of the premises, so the theory of statistical inference has to be based on the truth of the ingredients and this implies that one of the possible values for the quantity of interest is true. A theory of logical reasoning that could only determine falsity and never truth is not useful and similarly any valid measure of evidence must be able to indicate evidence in favor as well as evidence against.

Once a measure of statistical evidence is determined, an estimate of the quantity of interest is necessarily the value that maximizes the measure of evidence and the accuracy of the estimate can be assessed by looking at the size of the set of values that has evidence in their favor. The measure of evidence similarly necessarily determines whether there is evidence for or against a hypothesized value and the strength of this evidence can be assessed by comparison with the evidence associated with the other possible values for the quantity of interest.

Consider now requirement (I). If a satisfactory measure of evidence could be determined from the data alone, then this would be ideal, but currently this is not available and it is questionable whether it is even possible. It is *assumed* hereafter that the data x∈X can be regarded as having been produced by some probability distribution on the set X with unknown density f. If the data was collected via random sampling, then this assumption seems justified, but it is always an assumption. The density *f* is unknown and it is assumed that, once *f* is known, then this completely determines the answers to (E) and (H). The ingredients are then as follows: it is *assumed* that $f\in \{f_{\theta }:\theta \in \Theta \},$ a collection of densities on X indexed by the parameter θ∈Θ called the statistical model, and it is *assumed* that there is a probability measure Π with density π on Θ that represents beliefs of the investigator about the true value of θ∈Θ and called the prior. The ingredients correspond to the premises of a logical argument and these may be true or false.

It can be questioned as to whether both the model and prior are necessary for the development of a satisfactory theory and certainly minimizing the ingredients is desirable. However, as discussed in Section 3, it seems that a valid definition of a measure of evidence requires both and again the challenge is open to develop a satisfactory measure of evidence that uses fewer ingredients. In particular, the use of a prior is often claimed to be inappropriate as it is subjective in nature and, as the goal of a scientific investigation is to be as objective as possible, the prior seems contrary to that. It needs to be recognized, however, that all ingredients to a statistical analysis beyond the data are subjective as they are chosen by the statistician. As discussed in Section 3.2, it is possible to check both the model and the prior against the (objective) data to determine whether or not reasonable choices have been made. This can be considered as analogous to checking the consistency of the premises in a logical argument. In addition, it is possible to check whether or not the chosen ingredients have biased the results so that the inferences obtained are in fact foregone conclusions, namely, could have been made without even looking at the data. It is our view that checking for bias and checking for conflict with the data go a long way towards answering criticisms concerning the subjectivity inherent in a statistical analysis. Another implication from (I) is that no ingredient can be added to a statistical analysis unless it can be checked against the data and, as such, this rules out the use of loss functions.

It is not clear how the ingredients are to be chosen and guidance needs to be provided for this. Not much has been written about how the model is to be chosen, but certainly something needs to be said to justify a specific choice as part of the statistical reasoning argument. Much more has been written about the selection of the prior and the position is adopted here that it is necessary to base this on a clearly stated elicitation algorithm, namely, a prescription for how an expert can translate knowledge into beliefs as expressed via the prior.

In summary, the desiderata for a theory of statistical reasoning include the following: a methodology for choosing a model, an elicitation algorithm for selecting a prior, methodology for assessing the bias in the ingredients chosen, model checking and checking for prior-data conflict procedures and a theory of inference based upon a measure of statistical evidence.

## 3. A Theory of Statistical Reasoning

Choosing and checking the ingredients logically comes before inference, but it is convenient to discuss these in reverse order.

### 3.1. Relative Belief Inferences

Consider now a statistical problem with ingredients the data d, a model $\{f_{\theta }:\theta \in \Theta \},$ a prior π and interest is in making inference about ψ=Ψ(θ) for Ψ:Θ→Ψ where no distinction is made between the function and its range to save notation. Initially, suppose that all the probability distributions are discrete. This is not really a restriction in the discussion, however, as if something works for inference in the discrete case but does not work in the continuous case, then it is our view that the concept is not being applied correctly or the mathematical context is just too general. For us, the continuous case is always to be thought of as an approximation to a fundamentally discrete context, as measurements are always made to finite accuracy, and the approximation arises via taking limits. Some additional comments on the continuous case are made subsequently.

As discussed in Section 2, the basic object of inference is the measure of evidence and what is wanted is a measure of the evidence that any particular value ψ∈Ψ is true. Based on the ingredients specified, there are two probabilities associated with this value, namely, the prior probability $\pi_{\Psi }\left(\psi \right)$, as given here by the marginal prior density evaluated at ψ, and the posterior probability $\pi_{\Psi }\left(\psi \;|\;d\right)$, as given here by the marginal posterior density evaluated at ψ. In certain treatments of inference, $\pi_{\Psi }\left(\psi \;|\;d\right)$ is taken as a measure of the evidence that ψ is the true value, but, for a wide variety of reasons, this is not felt to be correct and Example 1 provides a specific case where this fails. In addition, this measure suffers from the same basic problem of *p*-values, namely, there is no obvious dividing line between evidence for and evidence against. Moreover, it is to be noted that probabilities measure belief and not evidence. If we start with a large prior belief in ψ, then, unless there is a large amount of data, there will still be a large posterior belief even if it is false and, similarly, if we started with a small amount of belief. There is agreement, however, to use the *principle of conditional probability* to update beliefs after receiving information or data and this is to be regarded as the first principle of the theory of relative belief.

Thus, what is the evidence that ψ is the true value to be measured? Basic to this is the *principle of evidence*: if $\pi_{\Psi }\left(\psi \;|\;d\right)>\pi_{\Psi }\left(\psi \right),$ there is evidence in favor, as belief has increased, if $\pi_{\Psi }\left(\psi \;|\;d\right)<\pi_{\Psi }\left(\psi \right),$ there is evidence against as belief has decreased and if, $\pi_{\Psi }\left(\psi \;|\;d\right)=\pi_{\Psi }\left(\psi \right),$ there is no evidence either way. This principle has a long history in the philosophical literature concerning evidence. This principle does not provide a specific measure of evidence but at least it indicates clearly when there is evidence for or against, independent of the size of initial beliefs, and it does suggest that any reasonable measure of the evidence depends on the difference, in some sense, between $\pi_{\Psi }\left(\psi \right)$ and $\pi_{\Psi }\left(\psi \;|\;d\right),$ namely, evidence is measured by *change in belief* rather than belief. A number of measures of this change have been proposed (see [1] for a discussion), but, by far, the simplest and the one that has the nicest properties is the relative belief ratio

(2) $$RB_{\Psi }\left(\psi \;|\;d\right)=\pi_{\Psi }\left(\psi \;|\;d\right)/\pi_{\Psi }\left(\psi \right).$$

Thus, if $RB_{\Psi }\left(\psi \;|\;d\right)>1,$ there is evidence for ψ being the true value, if $RB_{\Psi }\left(\psi \;|\;d\right)<1,$ there is evidence against ψ being the true value and no evidence either way if $RB_{\Psi }\left(\psi \;|\;d\right)=1.$ The use of the relative belief ratio to measure the evidence is the third and final principle of the theory, which we call the *principle of relative belief*. The relative belief ratio can also be written as $RB_{\Psi }\left(\psi \;|\;d\right)=m\left(d\;|\;\psi \right)/m\left(d\right),$ where *m* is the prior predictive density of the data and m(·|ψ) is the conditional prior predictive density of the data given Ψ(θ)=ψ.

Another natural candidate for a measure of evidence is the Bayes factor $BF_{\Psi }\left(\psi \;|\;d\right)$ as this satisfies the principle of evidence, namely, BF(ψ|d)>(<,=)1 when there is evidence for (against, neither) ψ being the true value. The Bayes factor can be defined in terms of the relative belief ratio as $BF_{\Psi }\left(\psi \;|\;d\right)=RB_{\Psi }\left(\psi \;|\;d\right)/RB_{\Psi }\left({\left\{\psi \right\}}^{c}\;|\;d\right)$ but not conversely. Note that the relative belief ratio of a set A⊂Ψ is $RB_{\Psi }\left(A\;|\;d\right)=\Pi_{\Psi }\left(A\;|\;d\right)/\Pi_{\Psi }\left(A\right),$ where $\Pi_{\Psi },\Pi_{\Psi }\left(\cdot \;|\;d\right)$ are the prior and posterior probability measures of Ψ, respectively. It might appear that $BF_{\Psi }\left(\psi \;|\;d\right)$ is a comparison between the evidence for ψ being true with the evidence for ψ being false, but it is provable that $RB_{\Psi }\left(A\;|\;d\right)>1$ implies $RB_{\Psi }\left(A^{c}\;|\;d\right)<1$ and conversely, so this is not the case. In addition, as subsequently discussed, in the continuous case, it is natural to take $BF_{\Psi }\left(\psi \;|\;d\right)=RB_{\Psi }\left(\psi \;|\;d\right).$

The principle of relative belief leads to an ordering of the possible values for ψ as $\psi_{1}$ is preferred to $\psi_{2}$ whenever $RB_{\Psi }\left(\psi_{1}\;|\;d\right)>RB_{\Psi }\left(\psi_{2}\;|\;d\right)$ since there is more evidence for $\psi_{1}$ than $\psi_{2}.$ When Ψ(θ)=θ, this agrees with the likelihood ordering, but likelihood fails to provide such an ordering for general ψ. It is common to use the profile likelihood ordering even though this is not a likelihood ordering and this does not agree with the relative belief ordering. It is noteworthy that the relative belief idea is consistent in the sense that inferences for all ψ=Ψ(θ) are based on a measure of the change in prior to posterior probabilities.

The relative belief ordering leads immediately to a theory of estimation. Basing inferences on the evidence requires that the relative belief estimate be a value ψ(d) that maximizes $RB_{\Psi }\left(\psi \;|\;d\right)$ and typically such a value is unique so $\psi \left(d\right)=\arg\sup_{\psi \in \Psi }RB_{\Psi }\left(\psi \;|\;d\right)$. It is also necessary to say something about the accuracy of this estimate in an application. For this, a set of values containing ψ(d) is quoted and the “size” of the set is taken as the measure of accuracy. Again, following the ordering based on the evidence, it is necessary that the set take the form $\left\{\psi :RB_{\Psi }\left(\psi \;|\;d\right)>c\right\}$ for some constant $c\leq \sup_{\psi \in \Psi }RB_{\Psi }\left(\psi \;|\;d\right)$ since, if $RB_{\Psi }\left(\psi_{1}\;|\;d\right)\leq RB_{\Psi }\left(\psi_{2}\;|\;d\right),$ then $\psi_{2}$ must be included whenever $\psi_{1}$ is. However, what *c* should be used? It is perhaps natural to chose *c* so that $\left\{\psi :RB_{\Psi }\left(\psi \;|\;d\right)>c\right\}$ contains some prescribed amount of posterior probability, so the set is a γ-credible region. However, there are several problems with this approach. For what γ should be chosen? Even if one is content with some particular γ, say γ=0.95, there is the problem that the set may contain values ψ with $RB_{\Psi }\left(\psi \;|\;d\right)<1$ and such a value has been ruled out since there is evidence against such a ψ being true. It is argued in [1] that the *plausibility set* $Pl_{\Psi }\left(d\right)=\left\{\psi :RB_{\Psi }\left(\psi \;|\;d\right)>1\right\}$ be used as $Pl_{\Psi }\left(d\right)$ contains all the values for which there is evidence in favor of it being the true value. In general circumstances, it is provable that $RB_{\Psi }\left(\psi \left(d\right)\;|\;d\right)>1$ so $Pl_{\Psi }\left(x\right)\neq \phi .$ There are several possible measures of size, and certainly the posterior content $\Pi_{\Psi }\left(Pl_{\Psi }\left(d\right)\;|\;d\right)$ is one as this measures the belief that the true value is in $Pl_{\Psi }\left(d\right),$ but also some measure such as length or cardinality is relevant. If $Pl_{\Psi }\left(d\right)$ is small and $\Pi_{\Psi }\left(Pl_{\Psi }\left(d\right)\;|\;d\right)$ large, then ψ(d) can be judged to be an accurate estimate of ψ.

It is immediate that $RB_{\Psi }\left(\psi_{0}\;|\;d\right)$ is the evidence concerning $H_{0}:\Psi \left(\theta \right)=\psi_{0}.$ The evidential ordering implies that the smaller $RB_{\Psi }\left(\psi_{0}\;|\;d\right)$ is than 1, the stronger is the evidence against $H_{0}$ and the bigger it is than 1, the stronger is the evidence in favor $H_{0}$, but how is one to measure this strength? In [16], it is proposed to measure the *strength of the evidence* via

(3) $$\Pi_{\Psi }\left(\left.RB_{\Psi }\left(\psi \;|\;d\right)\leq RB_{\Psi }\left(\psi_{0}\;|\;d\right)\;\right|\;d\right),$$

which is the posterior probability that the true value of ψ has evidence no greater than that obtained for the hypothesized value $\psi_{0}.$ When $RB_{\Psi }\left(\psi_{0}\;|\;d\right)<1$ and (3) is small, then there is strong evidence against $H_{0}$ since there is a large posterior probability that the true value of ψ has a larger relative belief ratio. Similarly, if $RB_{\Psi }\left(\psi_{0}\;|\;d\right)>1$ and (3) is large, then there is strong evidence that the true value of ψ is given by $\psi_{0}$ since there is a large posterior probability that the true value is in $\left\{\psi :RB_{\Psi }\left(\psi \;|\;x\right)\leq RB_{\Psi }\left(\psi_{0}\;|\;d\right)\right\}$ and $\psi_{0}$ maximizes the evidence in this set. Additional results concerning $RB_{\Psi }\left(\psi_{0}\;|\;d\right)$ as a measure of evidence and (3) can be found in [1,16].

For continuous parameters, it is natural to define $RB_{\Psi }\left(\psi \;|\;d\right)=\lim_{\epsilon \to 0}RB_{\Psi }\left(N_{\epsilon }\left(\psi \right)\;|\;d\right),$ where $N_{\epsilon }\left(\psi \right)$ is a sequence of sets converging nicely to {ψ} as ϵ→0. When the densities are continuous at ψ, then this limit equals (2) so this is a measure of evidence in general circumstances. In addition, it is natural to define the Bayes factor by $BF_{\Psi }\left(\psi \;|\;d\right)=\lim_{\epsilon \to 0}BF_{\Psi }\left(N_{\epsilon }\left(\psi \right)\;|\;d\right)$ and, when the densities are continuous at ψ, then $BF_{\Psi }\left(\psi \;|\;d\right)=RB_{\Psi }\left(\psi \;|\;d\right).$

A variety of consistency results, as the amount of data increases, are proved in [1] concerning the estimation and hypothesis assessment procedures. In particular, when $H_{0}$ is false, then (2) converges to 0 as does (3). When $H_{0}$ is true, then (2) converges to its largest possible value (greater than 1 and often *∞*) and, in the discrete case (3) converges to 1. In the continuous case, however, when $H_{0}$ is true, then (3) typically converges to a U(0,1) random variable. This simply reflects the approximate nature of the inferences and is easily resolved by requiring that a deviation δ>0 be specified such that if dist$(\psi_{1},\psi_{2})<\delta ,$ where dist is some measure of distance determined by the application, then this difference is to be regarded as immaterial. This leads to redefining $H_{0}$ as $H_{0}=\{\psi :$dist$\left(\psi ,\psi_{0})<\delta \right\}$ and typically a natural discretization of Ψ exists with $H_{0}$ as one of its elements. With this modification (3) converges to 1 as the amount of data increases when $H_{0}$ is true. Given that data is always measured to finite accuracy, the value of a typical continuous-valued parameter can only be known to a certain finite accuracy no matter how much data is collected. Thus, such a δ always exists and it is part of an application to determine the relevant value (see Example 7 here, Al-Labadi, Baskurt and Evans [7] and Evans, Guttman and Li [17] for developments on determining δ). These results establish that, as the amount of data increases, relative belief inferences will inevitably produce the correct answers to estimation and hypothesis assessment problems.

It is immediate that relative belief inferences have some excellent properties. For example, any 1-1 increasing function of $RB_{\Psi }\left(\cdot \;|\;d\right),$ such as $\log RB_{\Psi }\left(\cdot \;|\;d\right),$ can be used to measure evidence as the inferences are invariant to this choice. In addition, $RB_{\Psi }\left(\cdot \;|\;d\right)$ is invariant under smooth reparameterizations and so all relative belief inferences possess this invariance property. For example, MAP (maximum a posteriori) inferences are not invariant and this leads to considerable doubt about their validity (see also Example 1). In [1], results from a number of papers are summarized establishing optimality results for relative belief inferences in the collection of all Bayesian inferences. For example, Al-Labadi and Evans [18] establish that relative belief inferences for ψ have optimal robustness to the prior $\pi_{\Psi }$ properties. In addition, as discussed in Section 3.2, since the inferences are based on a measure of evidence a key criticism of Bayesian methodology can be addressed, namely, the extent to which the inferences are biased can be measured.

Relative belief prediction inferences for future data are naturally produced by using the ratio of the posterior to prior predictive densities for the quantity in question. The following example illustrates this and demonstrates significant advantages for relative belief.

**Example** **1.** Prediction for Bernoulli sampling.

Consider an example discussed in Chapter 6 of [19] who further reference [9]. A tack is flipped with x=1 indicating the tack finishes point up and x=0 otherwise, so x∼ Bernoulli(θ). Suppose the prior is θ∼U(0,1) and the goal is to predict *f* future observations $\left(y_{1},\ldots ,y_{f}\right)$ having observed *n* independent tosses $\left(x_{1},\ldots ,x_{n}\right)$. The posterior of θ is beta$(n\bar{x}+1,n\left(1-\bar{x}\right)+1),$ the prior predictive density of $\left(x_{1},\ldots ,x_{n}\right)$ is mn(x1,…,xn)=1/(n+1)nnx¯ and the posterior predictive density for $\left(y_{1},\ldots ,y_{f}\right)$ is

(4) mn,f((y1,…,yf)|(x1,…,xn))=(n+1)nnx¯(n+f+1)n+f(n+f)nn+fx¯+fn+fy¯,

which is constant for all $\left(y_{1},\ldots ,y_{f}\right)$ with the same value of $\bar{y}.$ Maximizing (4) gives the MAP predictor of $(y_{1},\ldots ,y_{f}).$ If $n\bar{x}/\left(n+f\right)>1/2,$ then the maximum occurs at $\left(y_{1},\ldots ,y_{f}\right)$ with $\bar{y}=1,$ namely, $\left(y_{1},\ldots ,y_{f}\right)=\left(1,\ldots ,1\right).$ If $n\bar{x}/\left(n+f\right)<1/2,$ then the maximum occurs at $\left(y_{1},\ldots ,y_{f}\right)$ with $\bar{y}=0,$ namely, $\left(y_{1},\ldots ,y_{f}\right)=\left(0,\ldots ,0\right).$ If $n\bar{x}/\left(n+f\right)=1/2$, then a maximum occurs at both $\left(y_{1},\ldots ,y_{f}\right)=\left(0,\ldots ,0\right)$ and $\left(y_{1},\ldots ,y_{f}\right)=\left(1,\ldots ,1\right)$. Thus, using MAP gives the absurd result that $\left(y_{1},\ldots ,y_{f}\right)$ is always predicted to be either all 0s or all 1s. Clearly, there is a problem here with using MAP.

Now suppose $\left(x_{1},\ldots ,x_{n}\right)=\left(0,\ldots ,0\right)$ so the prediction is all 0s and

mn,f((y1,…,yf)|(0,…,0))=(n+1)/(n+f+1)n+ffy¯.

For fixed f, then $m_{n,f}\left(\left(y_{1},\ldots ,y_{f}\right)\;|\;\left(0,\ldots ,0\right)\right)\to 0$ as n→∞ whenever $\bar{y}\neq 0$ and converges to 1 when $\bar{y}=0$. Diaconis and Skyrms [19] note, however, that when f=n, then $m_{n,n}\left(\left(0,\ldots ,0\right)\;|\;\left(0,\ldots ,0\right)\right)\to 1/2$ as n→∞ and make the comment “If this is an unwelcome surprise, then perhaps the uniform prior is suspect.” They also refer to some attempts to modify the prior to avoid this phenomenon, which clearly violates an essential component of the Bayesian approach. In our view, there is nothing wrong with the uniform prior, rather the problem lies with using posterior probabilities implicitly as measures of evidence, both to determine the predictor and to assess its reliability.

The relative belief ratio for $\left(y_{1},\ldots ,y_{f}\right)$ is

(5) RB((y1,…,yf)|(x1,…,xn))=(n+1)nnx¯(f+1)ffy¯(n+f+1)n+f(n+f)nn+fx¯+fn+fy¯.

**Figure 1:**
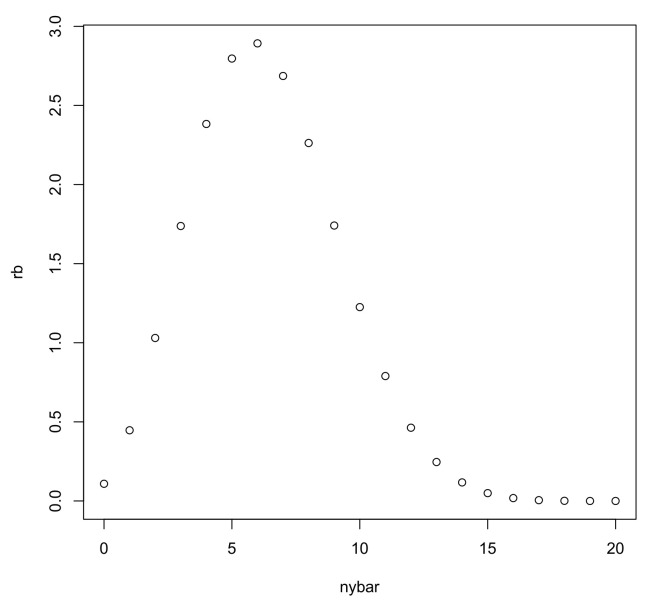
Plot of the relative belief ratio when $n=20,n\bar{x}=6$ in Example 1.

With n=f=20 and $n\bar{x}=6,$ Figure 1 gives the plot of (5) as a function of $n\bar{y}.$ The best relative belief predictor of $\left(y_{1},\ldots ,y_{f}\right)$ is any sample with $f\bar{y}=6$ and $Pl_{n}\left(x_{1},\ldots ,x_{n}\right)=\left\{\left(y_{1},\ldots ,y_{f}\right):f\bar{y}=2,3,\ldots ,10\right\}$ has posterior content 0.893. Thus, there is reasonable belief that the plausibility set contains the “true” future sample but certainly there are many such samples. By contrast with MAP, a sensible prediction is made using relative belief.

For the case when f=n and $\left(x_{1},\ldots ,x_{n}\right)=\left(0,\ldots ,0\right),$ then

RB((y1,…,yn)|(0,…,0))=(n+1)2(2n+1)nny¯2nny¯=(n+1)2(2n+1)∏i=0n−12−y¯−i/n(2−i/n),

which is decreasing in $\bar{y}$ and so is maximized for the sample with $\bar{y}=0.$ Similarly, when $\bar{x}=1$, the predictor is the sample with $\bar{y}=1.$ Thus, at the extremes, the predictions based on MAP and relative belief are the same, but otherwise there is a sharp disagreement. In addition,

$$Pl_{n}\left(0,\ldots ,0\right)=\left\{\left(y_{1},\ldots ,y_{n}\right):RB\left(\left(y_{1},\ldots ,y_{n}\right)\;|\;\left(0,\ldots ,0\right)\right)>1\right\}$$

always contains $\left(y_{1},\ldots ,y_{n}\right)=\left(0,\ldots ,0\right)$ and for any c∈(0,1] such that $\bar{y}\geq c,$

$$\begin{array}{c} RB\left(\left(y_{1},\ldots ,y_{n}\right)\;|\;\left(0,\ldots ,0\right)\right)=\left\{{\left(n+1\right)}^{2}/\left(2n+1\right)\right\}\prod_{j=0}^{n-1}\left(1-\bar{y}/\left(2-j/n\right)\right)\\ \leq \left\{{\left(n+1\right)}^{2}/\left(2n+1\right)\right\}{\left(1-c/2\right)}^{n}\to 0 \end{array}$$

as n→∞. Therefore, for any c∈(0,1], there is an N, such that for all n>N, then $Pl_{n}\left(0,\ldots ,0\right)$ contains no $\left(y_{1},\ldots ,y_{n}\right)$ having a proportion of 1s that is *c* or greater. Thus, $Pl_{n}\left(0,\ldots ,0\right)$ is shrinking as *n* increases in the sense that it contains only samples with smaller and smaller proportion of 1s as *n* increases.

The posterior content of the plausibility region equals

(6) ∑{ny¯:RB((y1,…,yn)|(0,…,0))>1}mn,f((y1,…,yf)|(0,…,0))nny¯,

which equals the sum over all the summands that are greater than 1/(n+1) and

mn,f((y1,…,yf)|(0,…,0))nny¯=(n+1)(2n+1)∏i=0n−12−y¯−i/n(2−i/n)=(n+1)(2n+1)1y¯=012y¯=1n121−1/n2−1/ny¯=2n⋮⋮121−1/n2−1/n⋯1−(k−1)/n2−(k−1)/ny¯=kn⋮⋮.

When $\bar{y}=k/n$, the corresponding term converges to ${\left(1/2\right)}^{k+1}.$ Thus, for all *n* large enough, the sum (6) contains the terms for $\bar{y}=0,1/n,\ldots ,k/n.$ Therefore, for ϵ>0 and all *n* large enough, (6) is greater than $\left(1/2\right)\left[1+1/2+\cdots +{\left(1/2\right)}^{k}\right]-\epsilon =1-{\left(1/2\right)}^{k+1}-\epsilon$ and the posterior content of $Pl_{n}\left(0,\ldots ,0\right)$ converges to 1.

Thus, relative belief also behaves appropriately when f=n and $\bar{x}=0$ while MAP does not. The failure of MAP might be attributed to the requirement that the entire sample $\left(y_{1},\ldots ,y_{n}\right)$ be predicted. If instead it was required only to predict the value $n\bar{y},$ then the prior predictive of this quantity is uniform on {0,1,…,f}, the posterior of $n\bar{y}$ equals $RB\left(\left(y_{1},\ldots ,y_{f}\right)\;|\;\left(x_{1},\ldots ,x_{n}\right)\right)/\left(f+1\right)$ and the relative belief ratio for $n\bar{y}$ equals $RB(\left(y_{1},\ldots ,y_{f}\right)\;|\;\left(x_{1},\ldots ,x_{n}\right)).$ Thus, as is often the case when the quantity in question has a uniform prior, MAP and relative belief estimates are the same. However, even in this case, there is no natural cut-off for MAP inferences to say when there is evidence for or against a particular value. The fact that it is necessary to modify the problem in this way to get a reasonable inference is, in our view, a substantial failing of MAP. It seems reasonable to suggest that, when an inference approach is shown to perform poorly on such examples, that it not be generally recommended. Additional examples of poor performance of MAP are discussed in [1].

It is notable that, while the relative belief approach to inference has been described here using statistical models and priors, in reality, everything can be cast in terms of a single probability model however such an object arises. Thus, if *P* is a probability measure on a sample space Ω and A⊂Ω is an event whose truth value is unknown but C⊂Ω is known to be true, then the evidence concerning the truth of *A* is given by RB(A|C)=P(A|C)/P(A), with this defined by the appropriate limit when either *A* or *C* is a null event. As discussed in [1], the relative belief approach to inference can be seen as essentially probability theory together with the principles of evidence and relative belief.

### 3.2. Choosing and Checking the Ingredients

The first choice that must be made is the model and there are a number of standard models used in practice. There is not a lot written about this step, however, and yet it is perhaps the most important step in solving a statistical problem. It is generally accepted that the correct way to choose a prior is through elicitation. This means that a methodology is prescribed that directs an expert in the application area on how to translate their knowledge into a prior. There are various default priors in use that avoid this elicitation step, but it is far better to recommend that sufficient time and energy be allocated for the elicitation of a proper prior. Staying within the context of probability suggests that a variety of paradoxes and illogicalities are avoided.

Given the ingredients, the relative belief inferences may be applied correctly, but it is still reasonable to ask if these ingredients are appropriate for the particular application. If not, then the inferences drawn cannot be considered valid. There are at least two questions about the ingredients that need to be answered, namely, is there bias inherent in the choice of ingredients and are the ingredients contradicted by the data?

The concern for bias is best understood in terms of assessing the hypothesis $H_{0}:\Psi \left(\theta \right)=\psi_{0}.$ Let M(·|ψ) denote the prior predictive distribution of the data given that Ψ(θ)=ψ. Bias against $H_{0}$ means that the ingredients are such that, with high probability, evidence will not be obtained in favor of $H_{0}$ even when it is true. Bias against is thus measured by

(7) $$M(RB_{\Psi }\left(\psi_{0}\;|\;D\right)\leq 1\;|\;\psi_{0}).$$

If (7) is large, then obtaining evidence against $H_{0}$ seems like a foregone conclusion. For bias in favor of $H_{0}$, consider $M(RB_{\Psi }\left(\psi_{0}\;|\;D\right)\geq 1\;|\;\psi_{*})$ where dist$(\psi_{*},\psi_{0})=\delta ,$ so $\psi_{*}$ is a value that just differs from the hypothesized value by a meaningful amount. Bias in favor of $H_{0}$ is then measured by

(8) $$\underset{\psi_{*}\in \left\{\psi :\;\mathrm{dist}\left(\psi ,\psi_{0}\right)=\delta \right\}}{\sup}M\left(RB_{\Psi }\left(\psi_{0}\;|\;D\right)\geq 1\;|\;\psi_{*}\right).$$

If (8) is large, then obtaining evidence in favor of $H_{0}$ seems like a foregone conclusion. Typically, $M(RB_{\Psi }\left(\psi_{0}\;|\;D\right)\geq 1\;|\;\psi_{*})$ increases as dist$\left(\psi_{*},\psi_{0}\right)$ increases so (8) is an appropriate measure of bias in favor of $H_{0}$. The choice of the prior can be used somewhat to control bias but typically a prior that makes one bias lower just results in making the other bias higher. It is established in [1] that, under quite general circumstances, both biases converge to 0 as the amount of data increases. Thus, bias can be controlled by design a priori.

The model needs to be checked against the data for, if the data *d* lies in the tails of every distribution in the model, then this suggests model failure. There are a wide variety of approaches to assessing this and these are not reviewed here. One relevant comment is that, at this time, there do not seem to exist general methodologies for modifying a model when model failure is encountered.

The prior can also be checked for conflict with the data. A conflict means that the observed data are in the tails of all those distributions in the model where the prior primarily places its mass. For a minimal sufficient statistic *T* for the model, Evans and Moshonov [20] used the tail probability

(9) $$M_{T}\left(m_{T}\left(t\right)\leq m_{T}\left(T\left(d\right)\right)\right)$$

to assess prior-data conflict where (9) small indicates prior-data conflict. In [21], it is shown that, under general circumstances, (9) converges to $\Pi (\pi \left(\theta \right)\leq \pi \left(\theta_{true}\right))$ as the amount of data increases. There are a variety of refinements of (9) that allow for looking at particular components of a prior to isolate where a problem with the prior may be. In [22], a method is developed for replacing a prior when a prior-data conflict has been detected. This does not mean simply replacing a prior by one that is more diffuse, however, as is demonstrated in Section 4.1.

## 4. Binary-Valued Response Regression Models

The following example, based on real data, is used to illustrate each aspect of the approach to statistical reasoning recommended here.

**Example** **2.** Bioassay experiment.

Table 1 gives the results of exposing animals to various levels in g/mL of a dosage of a toxin, where $x_{2}$ is the log-dosage and the number of deaths is recorded at each dosage (see [23]). The dosages range from $e^{-0.86}=0.423$ to $e^{0.73}=2.075$ g/mL. The logistic regression model $p\left(x_{1},x_{2}\right)=G\left(\beta_{1}+\beta_{2}x_{2}\right)$ is considered for this data, so $x_{1}\equiv 1,G\left(z\right)=e^{z}/\left(1+e^{z}\right),\left(\beta_{1},\beta_{2}\right)\in R^{2}$ and $p(1,x_{2})$ is the probability of death at dosage $x_{2}.$ The counts $T=(t_{1},t_{2},t_{3},t_{4})$ at the dosages comprise a minimal sufficient statistic for this problem with observed value (0,1,3,5). The conditional distribution of *T* given $\left(\beta_{1},\beta_{2}\right)$ is a product of binomials.

In [7], a goodness-of-fit test based on this data was applied for this model using a uniform prior on the space ${\left[0,1\right]}^{4}$ of all probabilities. Relative belief was used to assess the hypothesis that the model is correct and overwhelming evidence in favor of this model was obtained and so model correctness is assumed here. One goal is the estimation of $\left(\beta_{1},\beta_{2}\right)$ and another is the assessment of the hypothesis $H_{0}:\beta_{2}=0.$ Acceptance of $H_{0}$ implies that there is no relationship between the response and the predictor.

### 4.1. Eliciting the Prior

**Figure 2:**
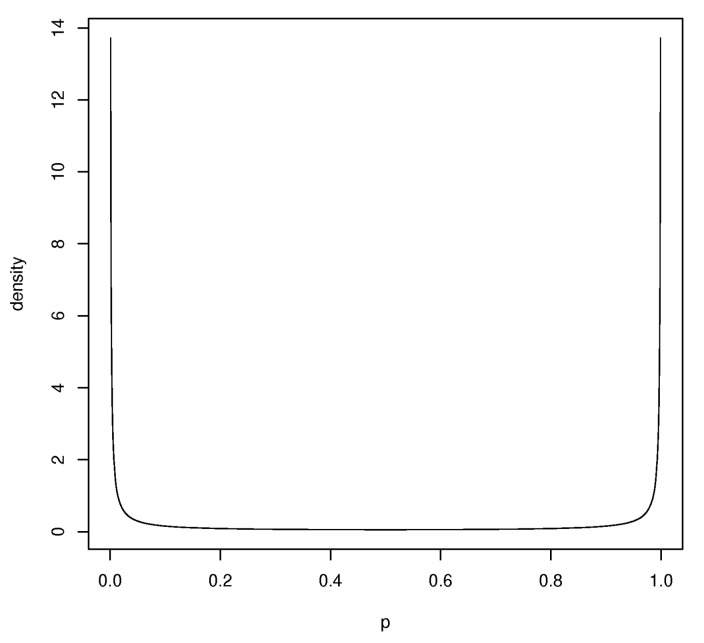
Prior density of of $p\left(1,x\right)=G\left(\beta_{1}+\beta_{2}x\right),$*G* is the standard logistic cdf, $\beta_{1},\beta_{2}∼N\left(0,20^{2}\right)$ and x=1.

Elicitation of a prior can be difficult when the interpretation of the parameters is unclear. For example, with model (1), it is not clear what the $\beta_{i}$ represents in contrast to linear models, where they represent either location parameters or rates of change with respect to predictors. This leads to attempts to put default priors on these quantities and there are problems with this approach. For example, suppose $p\left(1,x\right)=G\left(\beta_{1}+\beta_{2}x\right),$ where *G* is the standard logistic cdf and the prior is given by the $\beta_{i}$ being i.i.d. $N(0,\sigma^{2}),$ where $\sigma^{2}$ is chosen large to reflect little information about these values. In Figure 2, we have plotted the prior this induces on p(1,1) when σ=20. This reflects the fact that as σ grows all the prior probability for p(1,x) piles up at 0 and 1 and so this is clearly a poor choice and it is certainly not noninformative.

The strange behavior of diffuse normal priors has been noted by others. Bedrick, Christensen and Johnson [24,25], based on [26], make the recommendation that priors should instead be placed on the $p(x_{i}),$ as these are parameters for which there is typically prior information. Their recommendation is that *k* of the $x_{i}$ values be selected and then beta$\left(\alpha_{1i},\alpha_{2i}\right)$ priors be placed on the corresponding $p(x_{i})$ via eliciting prior quantiles. This results in more sensible priors but depends on the choice of the observed predictors, and it is unclear what kind of priors this induces on the $\beta_{i}.$

Following [24,25], priors here are elicited for the probabilities, but the approach is different. First, it is not required that the elicitation be carried out at observed values of the predictors. Rather, it is supposed that there is a set of linearly independent predictor vectors $w_{1},\ldots ,w_{k}$ where bounds can be placed on the probabilities in the sense that $l\left(w_{i}\right)\leq p\left(w_{i}\right)\leq u\left(w_{i}\right)$ for i=1,…,k with virtual certainty. By virtual certainty, it is meant that, for prior probability measure Π, then

(10) $$\Pi (l\left(w_{i}\right)\leq p\left(w_{i}\right)\leq u\left(w_{i}\right)\;\mathrm{for}\;i=1,\ldots ,k)\geq \gamma ,$$

where γ is chosen to be close to 1. For example, γ=0.99 certainly seems satisfactory for many applications, but a higher or lower standard can be chosen. The motivation for this is that typically information will be available for the probabilities such as it is known that $p(w_{i})$ is very small (or very large) or almost certainly that $p(w_{i})$ is in some specific range. Of course, for some of the $w_{i}$, virtually nothing may be known about $p(w_{i})$ and in that case taking $\left[l\left(w_{i}\right),u\left(w_{i}\right)\right]=\left[0,1\right]$ is appropriate. One implication of this is that when the choice is made $\left[l\left(w_{i}\right),u\left(w_{i}\right)\right]=\left[0,1\right]$ for every i, then the elicitation procedure should lead to a Π that is at least approximately uniform on the probabilities. The approach to elicitation, via stating bounds on parameter values that hold with virtual certainty, has been successfully employed in [2] to determine a prior for the multivariate normal model, and [17] to determine a prior for the multinomial model.

Another reason for allowing the elicitation procedure to be independent of the observed $x_{i}$ is that prior beliefs about $p(x_{i})$ may apply equally well about $p(x_{j})$ for some *j* simply because $x_{i}$ and $x_{j}$ are close, and then it seems that the correlation between the beliefs should be part of the prior. Modelling such correlations is harder and hopefully can be avoided by choosing the $w_{i}$ carefully. For example, requiring the $w_{i}$ to be mutually orthogonal seems like an appropriate way of achieving independence in many contexts.

The second way in which our approach differs from previous developments is that Π is restricted to the family of multivariate normal priors on β as this allows us to see directly how (10) translates into information about β. Note that (10) is equivalent to

(11) $$\begin{array}{c} \Pi (G^{-1}\left(l\left(w_{i}\right)\right)\leq G^{-1}\left(p\left(w_{i}\right)\right)\leq G^{-1}\left(u\left(w_{i}\right)\right)\;\mathrm{for}\;i=1,\ldots ,k)\\ =\Pi (G^{-1}\left(l\left(w_{i}\right)\right)\leq w_{i}^{\prime }\beta \leq G^{-1}\left(u\left(w_{i}\right)\right)\;\mathrm{for}\;i=1,\ldots ,k)\\ =\Pi (G^{-1}\left(l\left(W\right)\right)\leq W\beta \leq G^{-1}\left(u\left(W\right)\right))\geq \gamma , \end{array}$$

where $W={\left(w_{1}\ldots w_{k}\right)}^{\prime }\in R^{k\times k},l\left(W\right)={\left(l\left(w_{1}\right),\ldots ,l\left(w_{k}\right)\right)}^{\prime }\in R^{k},u\left(W\right)={\left(u\left(w_{1}\right),\ldots ,u\left(w_{k}\right)\right)}^{\prime }\in R^{k}.$ Thus, if $W\beta ∼N_{k}\left(\mu_{0},\Sigma_{0}\right),$ then $\beta ∼N_{k}\left(W^{-1}\mu_{0},W^{-1}\Sigma_{0}{\left(W^{-1}\right)}^{\prime }\right)$ and it is clear what this says about β.

The task then is to determine $\left(\mu_{0},\Sigma_{0}\right)$ so that (11) is satisfied. A natural choice for $\mu_{0}$ is to put $\mu_{0}=G^{-1}\left(c\left(W\right)\right)$ where c(W)=(l(W)+u(W))/2 is the centroid of the *k*-cell [l(W),u(W)]. For example, when $\left[l\left(W\right),u\left(W\right)\right]={\left[0,1\right]}^{k},$ then $c\left(W\right)=1_{k}/2,$ where $1_{k}$ is the *k*-dimensional vector of ones, which implies $\mu_{0}=0.$ Other choices for $\mu_{0}$ can be made if there are good reasons for this.

Given that the $w_{i}$ have been chosen so that prior beliefs about the probabilities $p(w_{i})$ are independent, this implies that the coordinates of Wβ are independent and so $\Sigma_{0}=\;$ diag$\left(\sigma_{1}^{2},\ldots ,\sigma_{k}^{2}\right)$ for some choice of the prior variances $\sigma_{i}^{2}.$ There are, however, typically many choices satisfying (11). For example, taking $\sigma_{i}^{2}=0$ for all *i* achieves this, but clearly this choice does not reflect what is actually known about the probabilities. As might be expected, the choice of the $\sigma_{i}^{2}$ is critical and dependent on G. Furthermore, as Figure 1 demonstrates, an injudicious choice results in absurdities.

Since $G^{-1}\left(u\left(w_{i}\right)\right)-\mu_{0i}>0$ and $G^{-1}\left(l\left(w_{i}\right)\right)-\mu_{0i}<0,$ both these values are infinite iff $\left[l\left(w_{i}\right),u\left(w_{i}\right)\right]=\left[0,1\right]$ and so no information is being introduced via the prior. In such a case, a uniform [0,1] prior on the probability results and the appropriate normal distribution is determined by approximating the distribution function *G* by a normal cdf (see Examples 3–5). Suppose then that at least one of $G^{-1}\left(u\left(w_{i}\right)\right)$ and $G^{-1}\left(l\left(w_{i}\right)\right)$ is finite and so $\sigma_{i}$ satisfies

(12) $$\Phi \left(\frac{G^{-1}\left(u\left(w_{i}\right)\right)-\mu_{0i}}{\sigma_{i}}\right)-\Phi \left(\frac{G^{-1}\left(l\left(w_{i}\right)\right)-\mu_{0i}}{\sigma_{i}}\right)\geq \gamma^{1/k},$$

as then independence ensures that (11) is satisfied. When both $G^{-1}\left(u\left(w_{i}\right)\right)$ and $G^{-1}\left(l\left(w_{i}\right)\right)$ are finite, the left side of (12) has the value 1, when $\sigma_{i}=0,$ is strictly decreasing to the value 0 as $\sigma_{i}\to \infty$ and so there are always values of $\sigma_{i}\geq 0$ satisfying (12). When both $G^{-1}\left(u\left(w_{i}\right)\right)$ and $G^{-1}\left(l\left(w_{i}\right)\right)$ are finite, there is a unique largest solution to (12), which is the preferred solution as it best represents the prior information, and it is easily found numerically by bisection. If $u(w_{i})=1$ and $l\left(w_{i}\right)\in \left(0,1\right),$ then $\sigma_{i}=\left(G^{-1}\left(l\left(w_{i}\right)\right)-\mu_{0i}\right)/\Phi^{-1}\left(1-\gamma^{1/k}\right)$ is the solution provided $\gamma >{\left(1/2\right)}^{k}$, which is a very weak requirement as recall that γ represents virtual certainty. If $u\left(w_{i}\right)\in \left(0,1\right)$ and $l(w_{i})=0,$ then $\sigma_{i}=\left(G^{-1}\left(u\left(w_{i}\right)\right)-\mu_{0i}\right)/\Phi^{-1}\left(\gamma^{1/k}\right)$ is the solution again provided $\gamma >{\left(1/2\right)}^{k}.$

The following examples consider the situation $l\left(w_{i}\right))=0,u\left(w_{i}\right)=1.$ In this case, $\mu_{0i}=0$ and $G^{-1}\left(p\left(w_{i}\right)\right)$ will be distributed with cdf *G* when $p\left(w_{i}\right)∼U\left(0,1\right).$ Generally, this leads to a need to approximate *G* by a normal cdf to obtain a normal prior, although no approximation is required in Example 3.

**Example** **3.** *Probit regression.*

Here, G=Φ and so $G^{-1}\left(p\left(w_{i}\right)\right)∼N\left(0,1\right)$ when $p\left(w_{i}\right)∼U\left(0,1\right).$ As such, $\sigma_{i}=1$ and the standard normal distribution on $G^{-1}\left(p\left(w_{i}\right)\right)$ corresponds to no information about $p(w_{i})$. When there is no information about any of the $p(w_{i}),$ then $\beta ∼N_{k}\left(0,W^{-1}{\left(W^{-1}\right)}^{\prime }\right),$ which equals the $N_{k}\left(0,I\right)$ distribution whenever W is an orthogonal matrix. In general, however, a lack of information about the probabilities leads to a prior on β that is dependent on W, namely, dependent on the values of predictor variables corresponding to the probabilities.

**Example** **4.** *Logistic regression.*

**Figure 3:**
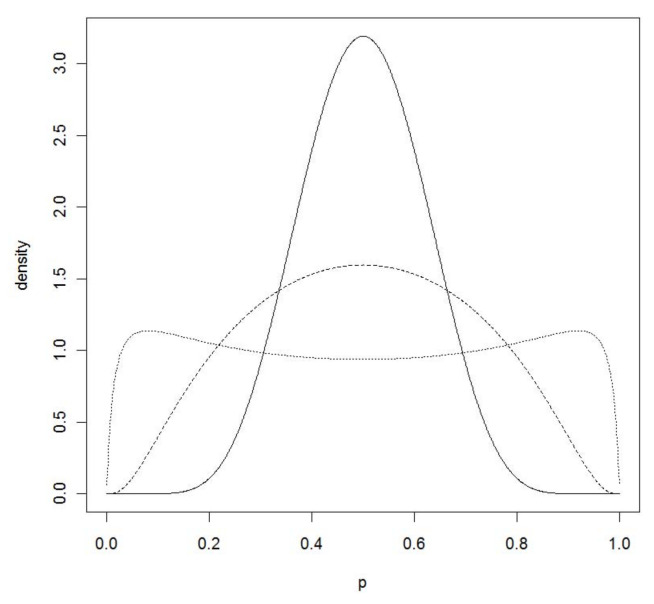
Plots of the density of $p=e^{z}/\left(1+e^{z}\right)$ when $z∼N(0,\lambda^{2})$ and λ=0.5 (–), λ=1.0 (- -), and λ=−1.702 (...).

In this case, *G* is the standard logistic cdf and so $w_{i}^{\prime }\beta =G^{-1}\left(p\left(w_{i}\right)\right)$ is distributed standard logistic when $p\left(w_{i}\right)∼U\left(0,1\right).$ A well-known $N(0,\lambda^{2})$ approximation to the standard logistic distribution, as discussed in [27], leads to normal priors that are much easier to work with. The optimal choice of λ, in the sense that it minimizes $\max_{x\in R^{1}}\left|\Phi \left(x/\lambda \right)-e^{x}/\left(1+e^{x}\right)\right|$ is given by λ=1.702, and this leads to a maximum difference less than 0.009. Clearly, this error will generally be irrelevant when considering priors for the probabilities in a logistic regression problem. Thus, when $w_{i}^{\prime }\beta ∼N\left(0,1.702^{2}\right)$, then $p(w_{i})$ is approximately distributed U(0,1) with the same maximum error. Figure 3 contains plots of the density of $p=e^{z}/\left(1+e^{z}\right)$ when $z∼N(0,\lambda^{2})$ for various choices of λ, and it is indeed approximately uniform when λ=1.702. Using normal probabilities rather than logistic probabilities leads to relatively small differences, so it seems reasonable to use a normal prior on β in a logistic regression.

**Example** **5.** t *regression.*

Suppose that *G* is taken to be the cdf of *t* with υ degrees of freedom. Table 2 presents the optimal choice of λ for a $N(0,\lambda^{2})$ approximation to the t(υ) cdf together with the maximum error. There does not appear to be much difference in using $t_{\upsilon }$ probabilities instead of normal ones unless υ is quite low.

Consider now an application of the elicitation algorithm.

**Example** **6.** *Bioassay experiment (Example 2 continued).*

In this example, k=2. To determine the prior, it is necessary to choose $W=(w_{1}$ $w_{2})\in R^{2\times 2}$ and [l(W),u(W)]. The authors are not experts in bioassay, but, given the range of dosages applied in the experiment, it is reasonable to suppose that an expert might be willing to put bounds on the probabilities that hold with prior probability γ=0.99 when $x_{2}=-0.50$ and $x_{2}=0.50$ leading to

$$W=\left(\begin{array}{cc} 1 & -1/2\\ 1 & 1/2 \end{array}\right).$$

Let us suppose that an expert believes with virtual certainty that the true probabilities lie in the intervals [0.15,0.75], when $x_{2}=-0.50,$ and in [0.25,0.95], when $x_{2}=0.50.$ Thus, the centroid of the 2-cell [0.15,0.75]×[0.25,0.95] is given by (0.45,0.60) and since $G^{-1}\left(p\right)=\log\left(p/\left(1-p\right)\right)$ for logistic regression, this implies $\mu_{0}=\left(G^{-1}\left(0.45\right),G^{-1}\left(0.60\right)\right)=\left(-0.20,0.41\right).$ In addition, $[G^{-1}\left(0.15\right),G^{-1}\left(0.75\right]=\left[-1.735,1.099\right]$ and $[G^{-1}\left(0.25\right),G^{-1}\left(0.95\right)]=\left[-1.099,2.944\right]$ so, using (12), the largest values of $\sigma_{1}$ and $\sigma_{2}$ satisfying $\Phi \left(1.299/\sigma_{1}\right)-\Phi \left(-1.535/\sigma_{1}\right)\geq {\left(0.99\right)}^{1/2}$ and $\Phi \left(2.534/\sigma_{2}\right)-\Phi \left(-1.509/\sigma_{2}\right)\geq {\left(0.99\right)}^{1/2}$ are given by $\sigma_{1}=0.490$ and $\sigma_{2}=0.580.$ Therefore, the prior on β is

(13) $$\begin{array}{c} \beta ∼N_{2}\left(W^{-1}{\left(-0.20,0.41\right)}^{\prime },W^{-1}\mathrm{diag}\left(0.490^{2},0.580^{2}\right){\left(W^{=1}\right)}^{\prime }\right)\\ =N_{2}\left(\left(\begin{array}{c} 0.105\\ 0.610 \end{array}\right),\left(\begin{array}{cc} 0.144 & 0.048\\ 0.048 & 0.577 \end{array}\right)\right). \end{array}$$

**Figure 4:**
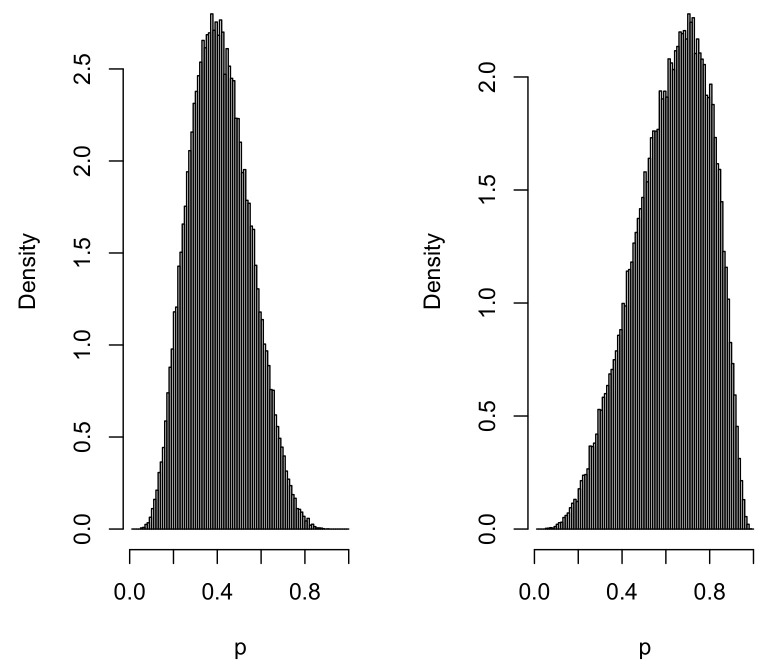
Density histograms of p(1,−0.8)(**left**) and p(1,0.8) (**right**) based on a sample of $10^{5}$ from the elicited prior in Example 6.

Figure 4 contains histograms of large samples from the priors on two extreme probabilities. The shape of the prior is similar for other values of $x_{2}.$

### 4.2. Measuring the Bias in a Prior

Consider applying the approach discussed in Section 3.2 to measuring bias in the prior derived in Section 4.1 for the bioassay example.

**Example** **7.** Bioassay experiment (Example 2 continued).

Consider whether or not there is bias induced by the prior in Example 6 with respect to the hypothesis $H_{0}:\beta_{2}=0.$ It is necessary to compute $M_{T}\left(RB_{2}\left(0\;|\;T\right)\leq 1\;|\;\beta_{2}=0\right),$ to measure bias against, and $\sup_{\beta_{2}\in \left\{-\delta ,\delta \right\}}M_{T}\left(RB_{2}\left(0\;|\;T\right)\geq 1\;|\;\beta_{2}\right),$ to measure bias in favor, where $RB_{2}\left(\cdot \;|\;T\right)$ is the relative belief ratio function for $\beta_{2}$ based on data *T* and δ>0 is such that, if $|\beta_{2}|<\delta ,$ then practically speaking $H_{0}$ is considered true. To determine δ, the more general problem of what changes in both $\beta_{1}$ and $\beta_{2}$ are deemed irrelevant is considered. Given the settings used in this experiment, it seems reasonable to consider $x_{2}$ as restricted to the interval [−1,1]. Then, whenever $|\beta_{1}-\beta_{1}^{\prime }|<\delta$ and $|\beta_{2}-\beta_{2}^{\prime }|<\delta ,$ the difference in log odds satisfies $|\beta_{1}+\beta_{2}x_{2}-\left(\beta_{1}^{\prime }+\beta_{2}^{\prime }x_{2}\right)|\leq 2\delta$, which implies that the ratio of the odds lies in $(e^{-2\delta },e^{2\delta }),$ which for small δ is approximately equal to (1−2δ,1+2δ). This in turn implies that the difference in the probabilities is less than 2δ. In this example, we take δ=0.01.

Now, RB(β1,β2|T)={∏i=14(5ti)pti(1,x2i)(1−p(1,x2i))5−ti}/mT(T) where

mT(T)=∫−∞∞∫−∞∞{∏i=14(5ti)pti(1,x2i)(1−p(1,x2i))5−ti}π(β)dβ.

The relative belief ratio for $\beta_{2}$ is $RB_{2}\left(\beta_{2}\;|\;T\right)=\int_{-\infty }^{\infty }RB\left(\beta_{1},\beta_{2}\;|\;T\right)\pi_{1}\left(\beta_{1}\;|\;\beta_{2}\right)\;d\beta_{1}=m_{T}\left(T\;|\;\beta_{2}\right)/m_{T}\left(T\right),$ where $\pi_{1}\left(\cdot \;|\;\beta_{2}\right)$ is the conditional prior density of $\beta_{1}$ given $\beta_{2},$ which (13) implies is the $N(0.105+0.083\left(\beta_{2}-0.610\right),0.140)$ distribution.

For given $T=(t_{1},t_{2},t_{3},t_{4}),$ the numerator and denominator in $RB_{2}\left(0\;|\;T\right)$ can be estimated via simulation but to calculate the biases we need to do this for many T. Consider the calculation of $M_{T}\left(RB_{2}\left(0\;|\;T\right)\leq 1\;|\;\beta_{2}=0\right)$ via the following Algorithm 1 and note that there are only $6^{4}=1296$ values of $\left(t_{1},t_{2},t_{3},t_{4}\right)\in {\left\{0,1,\ldots ,5\right\}}^{4}.$

**Algorithm 1:** Algorithm(i)simultaneously estimate the values $m_{T}\left(t_{1},t_{2},t_{3},t_{4}\right)$ for each $\left(t_{1},t_{2},t_{3},t_{4}\right)$ via a large sample from (13) and store these in a table,(ii)simultaneously estimate the values $m_{T}\left(t_{1},t_{2},t_{3},t_{4}\;|\;\beta_{2}=0\right)$ for each $\left(t_{1},t_{2},t_{3},t_{4}\right)$ via a large sample from $\pi_{1}\left(\cdot \;|\;0\right)$ and store these in a table,(iii)using the values in these two tables estimate $RB_{2}\left(0\;|\;T\right)$ for all values of *T* and then estimate $M_{T}\left(RB_{2}\left(0\;|\;T\right)\leq 1\;|\;\beta_{2}=0\right)$ by summing the $m_{T}\left(t_{1},t_{2},t_{3},t_{4}\;|\;\beta_{2}=0\right)$ for those $\left(t_{1},t_{2},t_{3},t_{4}\right)$ for which $RB_{2}\left(0\;|\;T\right)\leq 1.$

The bias in favor can be estimated at ±δ in exactly the same way but in step (ii) replace $\pi_{1}\left(\cdot \;|\;0\right)$ by $\pi_{1}\left(\cdot \;|\;-\delta \right)$ and by $\pi_{1}\left(\cdot \;|\;\delta \right)$. These computations were carried out and resulted in the bias against equaling 0.22 and the bias in favor equaling 0.77 at −δ and 0.78 at δ. Thus, there is some bias against $H_{0}$ with this prior, but there is appreciable bias in favor of $H_{0},$ at least when interest is in detecting deviations of size δ=0.01. For $\beta_{2}=5,$ however, the bias in favor of $H_{0}$ is 0.006, so there is in reality no bias in favor for large values of this parameter. One could contemplate modifying the prior to reduce the bias in favor at δ=0.01, but typically this just results in trading bias in favor with bias against. The real cure for excessive bias of either variety is to collect more data.

In general problems, the approach to the computations used here will not be feasible and so alternative methods are required. In certain examples, some aspects of the computations can be done exactly, but, in general, approximations such as those discussed in [28] will be necessary.

### 4.3. Checking and Modifying a Prior

Consider now checking the prior derived in Section 4.1 for the bioassay example.

**Example** **8.** *Bioassay experiment (Example 2 continued).*

The tail probability for checking the prior is given by

(14) $$M_{T}\left(m_{T}\left(t_{1},t_{2},t_{3},t_{4}\right)\leq m_{T}\left(0,1,3,5\right)\right).$$

As part of the algorithm discussed in Section 4.2, the values of $m_{T}\left(t_{1},t_{2},t_{3},t_{4}\right)$ have been estimated and the proportion of values of $m_{T}\left(t_{1},t_{2},t_{3},t_{4}\right)$ that satisfies the inequality gives the estimate of (14). In this example, (14) equals 0.41 so there is no prior-data conflict.

If prior-data conflict exists, the methods discussed in [21] are available to obtain a more weakly informative prior. In this case, it is necessary to be careful as it has been shown in Section 4.1 that simply increasing the variance of the prior will not necessarily accomplish this. On the other hand, there is the satisfying result that the $N_{2}\left(0,1.702^{2}I_{2}\right)$ prior, where $I_{2}$ is the identity matrix, will avoid prior-data conflict, so modifying the elicited prior to be closer to this prior is the appropriate thing to do when a conflict exists.

### 4.4. Inferences

Now, consider estimation and hypothesis assessment for the bioassay example.

**Example** **9.** *Bioassay experiment (Example 2 continued).*

Consider first the assessment of the hypothesis $H_{0}:\beta_{2}=0.$ From the algorithm, the quantity $RB_{2}\left(0\;|\;\left(0,1,3,5\right)\right)$ is available and this indicates whether there is evidence in favor of or against $H_{0}.$ In this case, $RB_{2}\left(0\;|\;\left(0,1,3,5\right)\right)=0.021$ so there is evidence against $H_{0}$. A calculation described below gives the value 0.001 for the strength, so it seems there is strong evidence against $H_{0}.$

To obtain the joint relative belief estimate of $(\beta_{1},\beta_{2}),$ it is necessary to maximize $RB(\beta_{1},\beta_{2}\;|\;T)$ as a function of $(\beta_{1},\beta_{2}),$ which is the same as the MLE. The plausibility region for this estimate is then $\left\{\left(\beta_{1},\beta_{2}\right):RB\left(\beta_{1},\beta_{2}\;|\;T\right)>1\right\}$ and the size and posterior content of this set provide a measure of the accuracy with which the coordinates of β can be simultaneously known. However, it is worth noting that the *i*-th coordinate of this joint estimate is not necessarily the value that has the greatest evidence in its favor, rather this is obtained by maximizing $RB_{i}\left(\beta_{i}\;|\;T\right)$ as a function of $\beta_{i}$ with plausibility region $\{\beta_{i}:RB_{i}\left(\beta_{i}\;|\;D\right)>1\}.$ Thus, the evidence approach dictates that $\beta_{i}$ be estimated by maximizing $RB_{i}\left(\beta_{i}\;|\;T\right).$ In problems where components of a multidimensional parameter are related by some constraint, then it is clearly necessary to estimate the components simultaneously, but this is not the case here.

The value of $RB_{i}\left(\beta_{i}\;|\;T\right)$ needs to be estimated and since this cannot be done for every value of $\beta_{i},$ its value is estimated on a finite grid. For this, let $\left[L_{i},U_{i}\right]$ be the effective prior support for $\beta_{i}$, say containing 0.995 of the probability, and form the grid $G_{i}=\left\{L_{i},L_{i}+\delta ,L_{i}+2\delta ,\ldots ,U_{i}-\delta ,U_{i}\right\}.$ For each $\beta_{1}\in G_{1}$ estimate $m_{T}\left(0,1,3,5\;|\;\beta_{1}\right)$ using a large sample from $\pi_{2}\left(\cdot \;|\;\beta_{1}\right)$ gives $RB_{1}\left(\beta_{1}\;|\;\left(0,1,3,5\right)\right)=m_{T}\left(0,1,3,5\;|\;\beta_{1}\right)/m_{T}\left(0,1,3,5\right).$ It is then easy to obtain the relative belief estimate $\beta_{1}\left(0,1,3,5\right)$ and plausibility region $\{\beta_{1}:RB_{1}\left(\beta_{1}\;|\;\left(0,1,3,5\right)\right)>1\}.$ The true relative belief estimate will differ from this estimate by at most δ, but this difference has been deemed irrelevant. A similar procedure is carried out for $\beta_{2}$ but now sampling from $\pi_{1}\left(\cdot \;|\;\beta_{2}\right)$ to estimate $m_{T}\left(0,1,3,5\;|\;\beta_{2}\right).$ The posterior density for $\beta_{i}$ satisfies $\pi_{i}\left(\beta_{i}\;|\;\left(0,1,3,5\right)\right)=RB_{i}\left(\beta_{i}\;|\;\left(0,1,3,5\right)\right)\pi_{i}\left(\beta_{i}\right)$ and, since $RB_{i}\left(\cdot \;|\;\left(0,1,3,5\right)\right)$ has been computed on the grids, these values can be used to approximate the contents of the plausibility regions via an obvious quadrature. Similarly, the strengths can be estimated and the strength quoted above equals $\sum_{\beta_{2}\in S\;}\pi_{2}\left(\beta_{2}\;|\;\left(0,1,3,5\right)\right)\delta$, where $S=G_{2}\cap \left\{\beta_{2}:RB_{2}\left(\beta_{2}\;|\;\left(0,1,3,5\right)\right)\leq RB_{2}\left(0\;|\;\left(0,1,3,5\right)\right)\right\}.$

**Figure 5:**
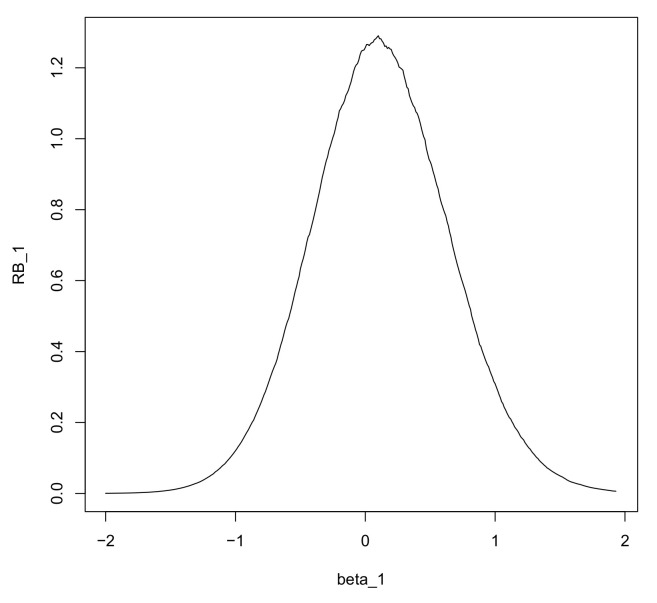
A plot of $RB_{1}\left(\cdot \;|\;\left(0,1,3,5\right)\right)$ over the effective support of the prior in Example 9.

Implementing this for $\beta_{1},$ the estimate $\beta_{1}\left(0,1,3,5\right)=0.11$ was obtained with plausibility region [−0.21,0.49] having posterior content 0.35. Thus, the range of plausible values for $\beta_{1}$ is not large, but there is not a high belief that the true value is in this interval. Figure 5 is a plot of $RB_{1}\left(\cdot \;|\;\left(0,1,3,5\right)\right).$

**Figure 6:**
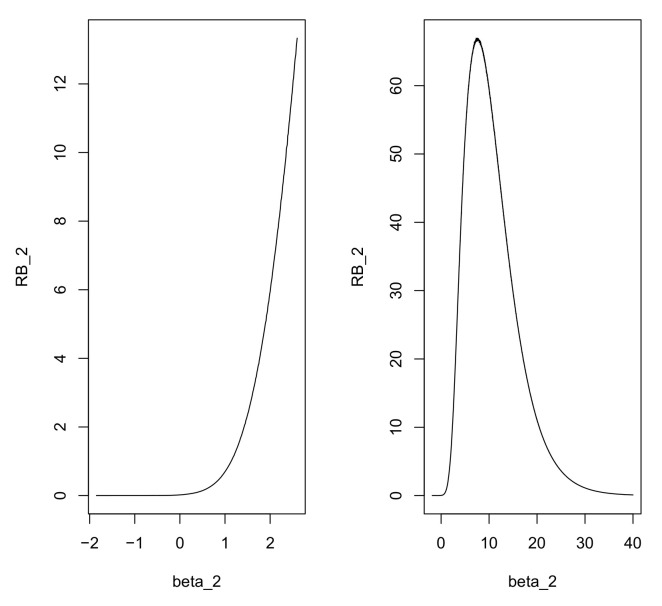
Plot of $RB_{2}\left(\cdot \;|\;\left(0,1,3,5\right)\right)$ over the effective support of the prior (**left panel**) and over a full range of possible values (**right panel**) in Example 9.

An interesting phenomenon occurs when considering the estimation of $\beta_{2}$. In Figure 6, the left panel plots $RB_{2}\left(\cdot \;|\;\left(0,1,3,5\right)\right)$ over the effective support of the marginal prior $\pi_{2}$ for $\beta_{2}.$ From this, it is clear that the relative belief estimate of $\beta_{2}$ lies outside this range. Recall, however, that the chosen prior passed the check for prior-data conflict. The check for prior-data conflict only tells us, however, that the observed data is consistent with at least some of the probabilities determined by where the prior places its mass. The right panel of Figure 6 is a plot of $RB_{2}\left(\cdot \;|\;\left(0,1,3,5\right)\right)$ over a much wider range. Note too that there is an important robustness property as shown in [18] for $RB_{2}\left(\cdot \;|\;\left(0,1,3,5\right)\right)$ as it is only weakly dependent on $\pi_{2}.$ In this case, $\pi_{2}$ does not place mass where it appears it should, but there is not enough data to detect the conflict. The relative belief estimate of $\beta_{2}$ is $\beta_{2}\left(0,1,3,5\right)=7.31$ and the plausibility region for $\beta_{2}$ is [1.14,30.48] with posterior content 0.83. As such, there is a great deal of uncertainty concerning the true value of $\beta_{2}.$

As long as it is possible to sample from the posterior for a 1-dimensional parameter, then the computations necessary for the inferences for such a parameter are feasible. As such, the Gibbs sampling algorithm of [29] is particularly relevant, although it is not needed in Example 9. The harder computations are those involving the various prior predictives, but these do not need to be highly accurate, as even one decimal place will indicate whether there is bias or prior-data conflict.

## 5. Conclusions

Criteria for a satisfactory theory of statistical reasoning have been developed. Perhaps more should be required, but it seems that those stated are necessary. In particular, the separation of the choosing and checking of the ingredients from the inference step has been emphasized as a key aspect based upon maintaining a desirable relationship with logical reasoning. An approach to statistical reasoning that satisfies these criteria has been presented. Application to a well-known example has shown that this approach can resolve anomalies/paradoxes that arise via commonly used methodology. Many other such instances of resolving inferential difficulties as well as results establishing optimal performance have been documented in [1]. An application of the approach to the problem of binary-valued response regression has been carried out, and it has been shown to lead to a number of novel insights and, in particular, a new elicitation algorithm has been developed for this problem.

## Figures and Tables

**Table 1:** Data in Example 1.

$x_{2}$	No. of Animals	No. of Deaths
−0.86	5	0
−0.30	5	1
−0.05	5	3
0.73	5	5

**Table 2:** Optimal choice of a $N(0,\lambda^{2})$ distribution to approximate a t(ν) distribution.

υ	30	20	10	5	2	1
λ	1.022	1.034	1.069	1.144	1.407	1.980
max error	0.002	0.003	0.006	0.013	0.031	0.058
